# Factors influencing practitioners’ who do not participate in ethically complex, legally available care: scoping review

**DOI:** 10.1186/s12910-021-00703-6

**Published:** 2021-09-28

**Authors:** Janine Brown, Donna Goodridge, Lilian Thorpe, Alexandra Hodson, Mary Chipanshi

**Affiliations:** 1 Faculty of Nursing, University of Regina, 111-116 Research Drive, Saskatoon, SK S7N 3R3 Canada; 2 College of Medicine, University of Saskatchewan, E1216, Health Sciences Building, 104 Clinic Place, Saskatoon, SK S7N 5E5 Canada; 3 Departments of Community Health and Epidemiology and Psychiatry, University of Saskatchewan, E3218, Health Sciences Building, 104 Clinic Place, Saskatoon, SK S7N 5E5 Canada; 4 Nursing Liaison Librarian, University of Regina Library, 3737 Wascana Parkway, Regina, SK S4S 0A2 Canada

**Keywords:** Conscientious objection, Medical ethics, Refusal to treat, Abstention, Care provision, Non-participation, Non-involvement

## Abstract

**Background:**

Evolving medical technology, advancing biomedical and drug research, and changing laws and legislation impact patients’ healthcare options and influence healthcare practitioners’ (HCPs’) practices. Conscientious objection policy confusion and variability can arise as it may occasionally be unclear what underpins non-participation. Our objective was to identify, analyze, and synthesize the factors that influenced HCPs who did not participate in ethically complex, legally available healthcare.

**Methods:**

We used Arksey and O’Malley’s framework while considering Levac et al.’s enhancements, and qualitatively synthesized the evidence. We searched Medline, CINAHL, JSTOR, EMBASE, PsychINFO, Sociological Abstracts, and ProQuest Dissertations and Theses Global from January 1, 1998, to January 15, 2020, and reviewed the references of the final articles. We included articles written in English that discussed the factors that influenced physicians and registered nurses (RNs) who did not participate in end-of-life (EOL), reproductive technology and health, genetic testing, and organ or tissue donation healthcare areas. Using Covidence, we conducted title and abstract screening, followed by full-text screening against our eligibility criteria. We extracted the article’s data into a spreadsheet, analyzed the articles, and completed a qualitative content analysis using NVivo12.

**Results:**

We identified 10,664 articles through the search, and after the screening, 16 articles were included. The articles sampled RNs (n = 5) and physicians (n = 11) and encompassed qualitative (n = 7), quantitative (n = 7), and mixed (n = 2) methodologies. The care areas included reproductive technology and health (n = 11), EOL (n = 3), organ procurement (n = 1), and genetic testing (n = 1). One article included two care areas; EOL and reproductive health. The themed factors that influenced HCPs who did not participate in healthcare were: (1) HCPs’ characteristics, (2) personal beliefs, (3) professional ethos, 4) emotional labour considerations, and (5) system and clinical practice considerations.

**Conclusion:**

The factors that influenced HCPs’ who did not participate in ethically complex, legally available care are diverse. There is a need to recognize conscientious objection to healthcare as a separate construct from non-participation in healthcare for reasons other than conscience. Understanding these separate constructs will support HCPs’ specific to the underlying factors influencing their practice participation.

## Background

Evolving medical technology, advancing biomedical and drug research, and changing laws and legislation impact patients’ healthcare options and influence healthcare practitioners’ (HCPs’) practices. In June 2016, Bill C-14 became law in Canada, which supported eligible patients’ right to access medical assistance in dying (MAID) [1]. This newly available end-of-life (EOL) healthcare option, cultivated interest at the convergence of HCPs’ care participation, conscientious objection, and patients’ access to care.

HCPs consider their care participation within the greater systems in which they practice. Healthcare delivery systems are regulated by federal and provincial law, influenced by local, regional, and national culture, and guided by employer policies. HCPs also practice within their professional codes of ethics and standards, and their individual moral values [2], and moral imperatives [3]. When navigating these considerations, some HCPs find their practices do not align with the care a patient requests and have a conscientious objection to care.

HCPs engage in conscientious objection (CO) when they decline to provide care because their participation is incompatible with their ethical, religious, or core moral beliefs [4]. Conscientious objection is a complex and sometimes polarizing topic of debate. Schuklenk (2015) noted that patients are “entitled” to receive care from HCPs because they became HCPs by voluntary choice [5]. Others contend that CO is unethical and constitutes an abandonment of professional obligation [6]. Weinstock (2014), however, posits that there are reasons to support a “limited right” CO in healthcare [7]. These reasons include that conscientious objection (1) provides HCPs the opportunity to reflect on their practice demands relative to their sense of self, (2) allows HCPs to deliberate complex moral issues and reflect on the laws, rules, and codes that regulate their practice, (3) accommodates the moral agency of HCPs with alternative views, and (4) fosters the examination of the underlying reasons for dissent [7]. Canadian HCPs’ professional codes of ethics address CO and non-abandonment of patients [8, 9]. However, a pan-Canadian review of CO policies noted “considerable potential for practitioner confusion exists based on the bewildering array of policies existing in many provinces and territories” and further noted significant variability in how to address conscience conflicts [10].

Conscientious objection policy confusion and variability can arise as it may occasionally be unclear what underpins non-participation. Dean [11] noted that HCPs’ non-participation may not always be precipitated by conscience, and Lachman [12] highlighted the need to distinguish conscience claims from non-participation influenced by cowardice, dislike, self-interest, discrimination, or prejudice. For instance, claims of conscience were noted in some care refusals that were based on HCPs' convenience, irrational fear, or reluctance to treat patients because of the patient’s unhealthy lifestyle choices [10]. Card [5] proposed that HCPs be required to declare their reasons for the objection. He explained this evaluation would assess the objection's reasonability and would ensure the non-participation (1) did not result in unreasonable harm to patients, (2) respected the power inequality between HCPs and patients, (3) was non-discriminatory, and (4) did not violate the duty of care. Shaw and Downie (2014) noted that confusion and variability surrounding CO could result in inconsistent patient care options and outcomes, increased healthcare costs, friction within the care team, and patient and provider uncertainty regarding care [10].

Our research project used a scoping review methodology. Scoping methodologies are useful for charting the relevant literature in an area of interest and exploring broad topics with multiple study designs [13]. Specifically, scoping reviews (1) examine the nature of the research activity in a given field, (2) determine the potential value of undertaking a full systematic review, (3) summarize and disseminate research findings, and (4) identify gaps in the existing research [13]. Using this methodology, we identified, analyzed, and synthesized the factors that influenced HCPs who do not participate in ethically complex, legally available care and further identified the research gaps to inform future areas of inquiry. The Arksey and O’Malley methodology framework [13], Levac et al.’s enhancements [14], and the PRISMA Scoping Review Checklist [15] were used to guide the research and its reporting. A protocol was published [16], and this paper reflects the final project.

This scoping review explored factors of conscience and non-conscience origins that influenced HCPs’ who do not participate in ethically complex, legally available healthcare. We considered ethically complex care as the care available in a morally pluralistic, evolving context with significant physical, mental, emotional, and social implications for patients, families, and healthcare providers. Specifically, our research question was, “What is known regarding the factors that influence physicians and registered nurses who do not participate in the ethically complex and legally available care areas of EOL (including assisted death), reproductive health and technology, genetic testing, and organ or tissue donation?” A team of five researchers conducted this project.

## Methods

### Identifying the relevant articles

The search protocol was developed by the team librarian and included MeSH, keywords, and synonyms (Appendix A). We chose these terms to capture the concepts broadly related to care non-participation. We accessed the Medline, CINAHL, JSTOR, EMBASE, PsychINFO, Sociological Abstracts, and ProQuest Dissertations and Theses Global databases and searched the period from January 1, 1998, to January 15, 2020. Our STARLITE [17] search strategy summary is in Table 1.

**Table 1:** STARLITE [17] literature search strategy summary

Sampling strategy	Comprehensive survey
Type of study	Any article that might contribute to answering the research question
Approaches	Electronic database searching and manually reviewing the reference lists of the articles that eventually met all inclusion/exclusion criteria
Range of Years	January 1, 1998, to January 15, 2020
Limits	Excluded grey literature and non-English articles
Inclusion/exclusion criteria	Per Table 2: Eligibility criteria for article selection
Terms used	Appendix A for initial literature search protocol
Electronic Databases	Medline, CINAHL, JSTOR, PsycINFO, ProQuest Dissertations and Theses Global, EMBASE and Sociological Abstracts

We completed a second search of the databases inclusive of non-English articles to thoroughly account for all articles relative to our project, which resulted in 1537 non-English articles. Given the abundance of identified articles, we did not search beyond our initial article inclusion date, and grey literature and non-English articles were excluded. We also reviewed their reference lists of the articles identified through the selection process to identify other potentially relevant articles.

### Article selection

Seeking to balance reasonable project boundaries within an extensive array of ethically complex, legally available care areas, we used our clinical and research experience to outline the article selection criteria and specify the included care areas (Table 2). We used Covidence [18] to organize and facilitate the article selection process. First, two team members evaluated each article’s title and abstract against the eligibility criteria. After screening a minimum of 30 studies, we cross-checked the screening results to support reliability in our understanding and application of the criteria. When we were satisfied with our cross-checking, we continued screening the articles. Articles with conflicted screening results were identified in Covidence, and subsequently, two team members determined their inclusion or exclusion by consensus. We refined the eligibility criteria prior to the full text-screening to support the exposition of the research question. Two team members then assessed the articles’ full-text, and again, the conflicted articles were discussed by two team members to determine their inclusion or exclusion by consensus. Article quality was not assessed, which was consistent with a scoping review methodology [13].

**Table 2:** Eligibility criteria for article selection

*Title and abstract eligibility criteria*	
Inclusion	Exclusion
Physicians and/or RNs in the sample, AND	Non-English studies, OR
Must include reasons or factors that precipitate or influence individual non-participation in legally available care, AND	Studies that included other health professional groups, OR
Must be within one of these healthcare areas: end-of-life care, reproductive technology, and health, genetic testing, organ or tissue donation, OR	Studies included nursing or medical students
The article speaks to the physician or RN CO in one of the identified healthcare areas	
*Additional full-text eligibility criteria*	
Additional inclusion	Additional exclusion
Must be a research study (as opposed to a theoretical discussion of constructs), AND	Conference abstract, OR
Care must be legally available where the study was conducted	The findings had aggregate results that included students or individuals other than RNs or physicians in the sample, OR
	The study included multiple jurisdictions, and the care is not legally available in all areas identified in the study, OR
	The care area was not identified

### Charting the data

As this scoping review formed part of a doctoral dissertation, the first author, supported by the second and third authors, led the data extraction, collation, and analysis. We populated the article’s information (including year, authors’ names, country, and journal), the article’s design (including methodology, objectives, care area, sample profession, and size), and the article’s findings into our data extraction spreadsheet. This spreadsheet was shared with all the research team members for cross-checking. With the support of NVivo 12 [19], the first author qualitatively analyzed the articles through open-coding and content analysis. Subsequently, through a process of code combining and refining, we developed a codebook and conducted thematic analysis [20]. All the team members had the opportunity to discuss and refine the interim and final findings.

### Patient and public participation

We did not involve patients or families in this research. However, as we are able, knowledge translation activities will occur to disseminate findings to knowledge users.

## Results

### Include and excluded articles

**Fig. 1:**
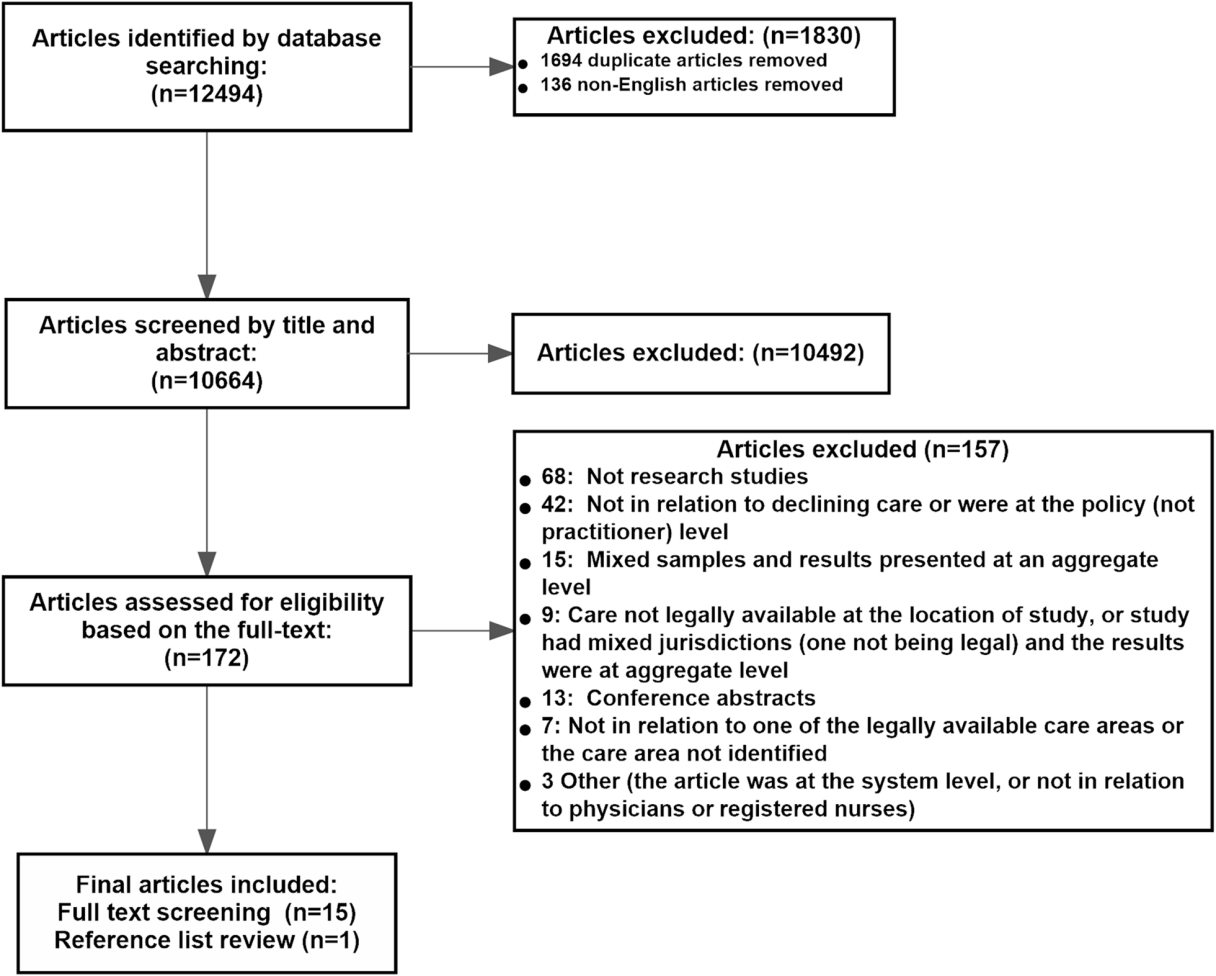
Study selection flow diagram

Through our literature search strategy, we identified 12,494 articles. In Covidence, we removed the duplicate and non-English articles that were not excluded through the database searches. Consequently, we had 10,664 articles available for the title and abstract screening. One hundred and seventy-two (172) articles remained after we applied our initial eligibility criteria, and 15 articles remained after we conducted the full-text screening against our refined eligibility criteria. We located one additional article by reviewing the reference lists of the included articles (Fig. 1).

### Analyzing the articles

The articles summary and contextual information are provided in Table 3 to frame our thematic findings. The included articles were published between 2000 and 2019, and eleven studies included physicians, and five included RNs. The articles originated in the United States (n = 8), Australia (n = 2), South Africa (n = 1), Brazil (n = 1), Norway (n = 1), Switzerland (n = 1), and Canada (n = 1). One article compared findings from the United States and the Netherlands. The articles comprised qualitative (n = 7), quantitative (n = 7), and mixed (n = 2) methodologies. The articles spanned multiple care areas and included reproductive health (n = 10), EOL care (including physician-assisted dying and medical assistance in dying) (n = 3), genetic testing (n = 1), and organ procurement (n = 1). One article included two care areas; EOL and reproductive health.

**Table 3:** Summary of included studies

First author	Year	Country	Methodology	Legally available care area	Profession	Sample size
Botes [29]	2000	South Africa	Qualitative	Reproductive health	RN	n = 1200 (open-ended questionnaire) and 22 focus groups
Bouthillier [34]	2019	Canada	Qualitative	Medical assistance in dying	Physician	n = 22 individual interviews
Clymin [32]	2012	Washington, USA	Mixed Methods (qualitative analysis of open text responses)	Physician-assisted dying	RN	n = 582
Curlin [31]	2008	Illinois, USA	Qualitative	Reproductive health	Physician	n = 19
Dawson [27]	2017	South Wales, Australia	Qualitative	Reproductive health	Physician	n = 28 and one focus group
Diniz [33]	2014	Brazil	Mixed Methods	Reproductive health	Physician	n = 1690 quantitative
						n = 50 qualitative
Escher [36]	2000	Switzerland	Quantitative	Genetic testing	Physician	n = 259 (response rate of 64%)
Harris [23]	2011	USA	Quantitative	Reproductive health	Physician	n = 1154 (response rate of 66%)
Holt [22]	2017	USA	Quantitative	Reproductive health	Physician	n = 744 (response rate of 29%)
Marek [24]	2004	California, USA	Quantitative	Reproductive health	RN	n-75 (response rate of 49%)
Nordberg [21]	2014	Norway	Qualitative	Reproductive health	Physician	n = 7 individual interviews
Seelig [25]	2006	USA	Quantitative	Reproductive health	Physician	n = 419 (response rate 53%)
Smith [35]	2017	Australia	Qualitative	Organ procurement	RN	n = 35 individual interviews
Stevens [28]	2017	Massachusetts, USA	Quantitative	End-of-Life, Physician-assisted dying, reproductive health	RN	n-297 (response rate 42%)
Tilburt [30]	2013	USA	Quantitative	Reproductive health^a^	Physician	n = 1032 (response rate 54%)
Willems [26]	2000	Oregon (USA) & Netherlands	Qualitative	End-of-Life, Physician-assisted dying, Euthanasia	Physician	n = 152 in Oregon
						n = 67 in the Netherlands

^a^Study included two care areas: Reproductive health and Euthanasia. As euthanasia is not legal in all US jurisdictions, data used from the reproductive health findings only

### Thematic findings

**Fig. 2:**
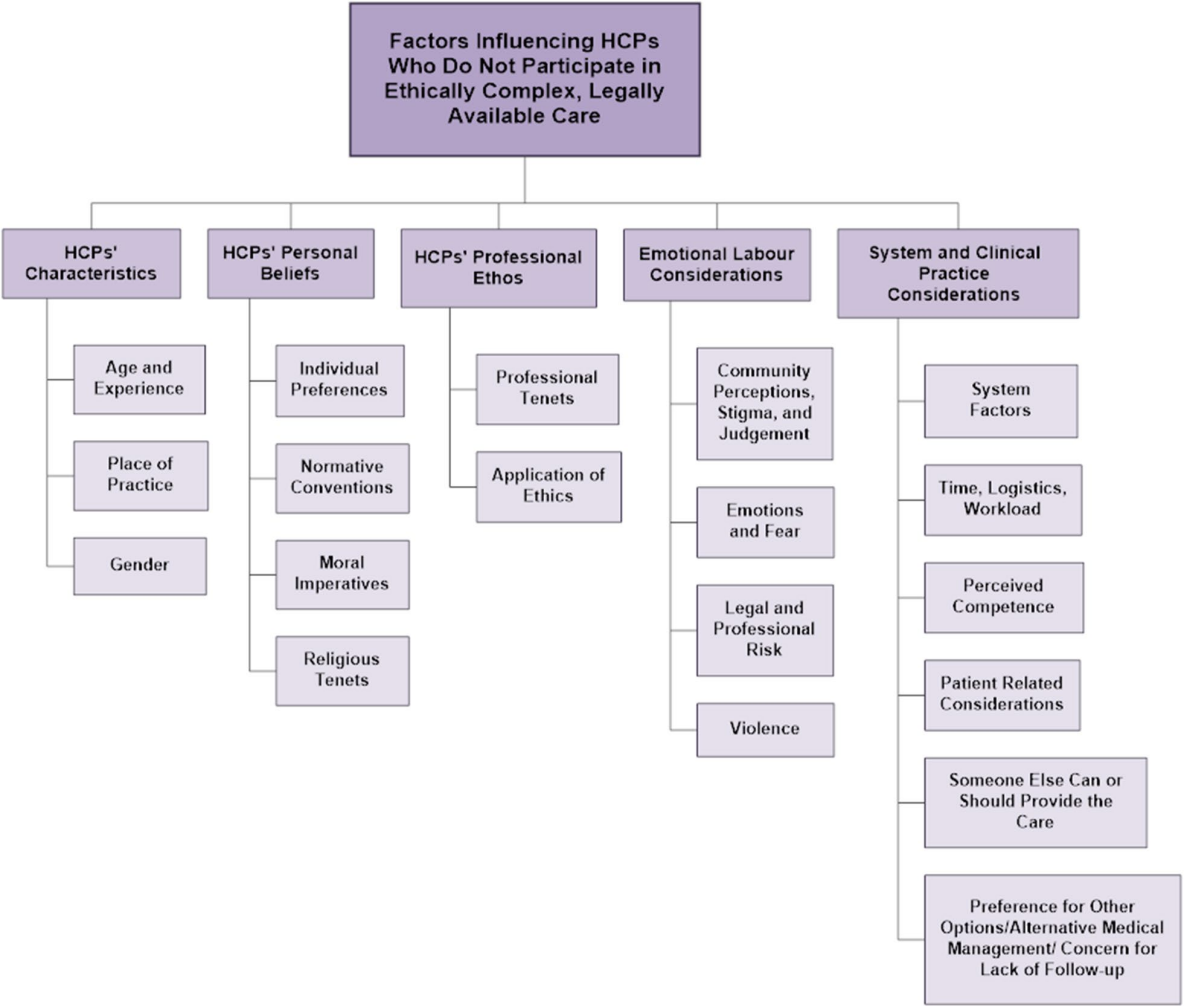
Thematic findings

We categorized the factors that influence HCPs who do not participate in ethically complex, legally available care into five themes. These themes are (1) HCPs' characteristics, (2) HCPs' personal beliefs, (3) HCPs' professional ethos (4), emotional labour considerations, and (5) system and clinical practice considerations (insert Fig. 2). Table 4 outlines the content-coding matrix, including the themed factors, the content codes, and the articles where the content code was applied.

**Table 4:** Included study content coding and themes

Themed factors	Content codes	The number of times the content code was applied	The article where the code was applied (by the first author)
HCPs’ characteristics	Age and experience	4	Harris, Holt, Marek, Nordberg
	Gender	3	Willems, Holt, Harris
	Place of practice	3	Harris, Holt, Seelig
HCPs’ personal beliefs	Individual preferences	6	Botes, Dawson, Stevens, Marek, Holt
	Normative conventions	3	Botes, Tilburt, Curlin
	Moral imperatives and conviction	8	Bouthillier, Clymin, Dawson, Tilburt, Nordberg, Marek, Smith, Diniz
	Religious tenets	13	Botes, Bouthillier, Clymin, Diniz, Harris, Holt, Nordberg, Tilburt, Willems, Curlin, Stevens, Smith, Dawson
HCPs’ professional ethos	Professional tenets	3	Botes, Bouthillier, Curlin
	Application of ethics	6	Escher, Holt, Nordberg, Curlin, Smith, Marek
Emotional labour considerations	Community perception, stigma, and judgement	4	Dawson, Smith, Bouthillier, Diniz
	Emotions and fear	3	Bouthillier, Dawson, Clymin
	Legal and professional risk	4	Bouthillier, Clymin, Diniz, Willems
	Violence	1	Seelig
System and clinical practice considerations	Perceived competence/lack of knowledge	5	Bouthillier, Clymin, Dawson, Holt, Smith
	Time, workload and logistics	3	Bouthillier, Dawson, Smith
	Preference for other care options, concern for lack of available follow-up care, alternative medical management	4	Escher, Harris, Bouthillier, Clymin
	Someone else can or should provide the care	6	Seelig, Botes, Clymin, Dawson, Holt, Nordberg
	System factors	4	Clymin, Dawson, Holt, Smith
	Patient-related considerations	6	Diniz, Harris, Holt, Marek, Willems, Curlin

#### HCPs’ characteristics

Age, years of experience, location of practice (including geographical region or clinical practice area), and gender were the identified characteristics of HCPs who do not participate in ethically complex, legally available care. One article highlighted that some HCPs developed opposition to care participation over time [21]. Conversely, other articles identified HCPs who had more experience [22], and HCPs who identified as “older” [23] were less likely to object to ethically complex, legally available care. Additionally, HCPs’ previous work experience specific to the care area influenced their care non-participation [24].

Non-participation in ethically complex, legally available care was more likely among HCPs who practiced in rural settings [23], and among HCPs who were located in the South or Midwest of the United States [22, 23]. Private practices (compared to hospital-based settings) [22, 25], and religiously affiliated practices (compared to non-religiously affiliated practices) [22] were associated with non-participation in ethically complex, legally available care, and non-participation was more likely among male HCPs [22, 23, 26].

#### HCPs’ personal beliefs

Personal beliefs influenced HCPs who did not participate in ethically complex, legally available care, and we coded these as individual preferences, normative conventions, moral imperatives or convictions, and religious tenets. HCPs' individual preferences were noted in the articles as “personal beliefs,” [27] “personal objections,” [22] “attitudes”, [24] “non-religious reasons”, [28] as care being an “unpleasant service”, [27] or as a “waste of taxpayers’ money”. [29] Normative conventions, or the socially and culturally shared notions about the way things are usually done [3], influenced HCPs’ non-participation in ethically complex, legally available care and were noted as HCPs’ consideration of rights and responsibilities [29], fairness [30], and if the request was counter to a “widely held societal norm” [31].

Non-participation was also influenced by a belief that the ethically complex, legally available care was fundamentally right or wrong [3], and we coded these as moral imperatives or convictions. Moral imperatives included “moral objections”, [27] “moral convictions”, [32] “moral duty”, [33] “moral beliefs”, [24, 34, 35] “sanctity”, [30] and that care refusal allowed HCPs “to be themselves” in care participation [21]. Lastly, some HCPs expressed care participation was counter to their religious tenets [21, 26–28, 30, 32–35], and identified that their participation would be “judged by God” [29] or would result in tensions between the HCPs’ beliefs and the patient’s care requests [31]. Specifically, HCPs who identified as Catholic, Protestant, Christian, Muslim, or who communicated the importance of religion were more likely to object to participation in ethically complex, legally available care [22, 23].

#### HCP’s professional ethos

Professional ethos influenced HCPs who do not participate in care. Some HCPs believed the care conflicted with the tenets of medicine [34] or the Hippocratic Oath [31]. Other HCPs noted the care misaligned with the promotion of health or the Nurses’ Pledge [29]. HCPs frequently noted that care participation was counter to their application of professional ethics [21, 22, 24, 31, 35, 36]. Specifically, this included beliefs that the care would “do more harm than good” [36], that the care would “harm the patient’s health” [22], and that HCPs had a “commitment to the patients medical good” [31].

#### Emotional labour considerations

Emotional labour, or the management of feelings [37], was considered by HCPs who do not participate in ethically complex, legally available care. Fear was documented as a primary emotional response in multiple articles [27, 32, 34]. Expressly HCPs: (1) feared the emotional aspects of care provision and its sequelae for the care provider [34]; (2) feared a potential backlash from others if they participated in care [27]; (3) feared patient death [34]; and/or (4) feared potential job loss [32]. Additionally, HCPs contemplated the risk of violence [25], the risk of medicolegal and/or professional repercussions [26, 32–34], and considered the stigma and judgment from their colleagues and the greater community [27, 33–35].

#### System and clinical practice considerations

System considerations influenced HCPs' non-participation in ethically complex, legally available care, including (1) “uncertainty about facility or professional policies” [32], (2) practices that “would not permit” the care option (i.e., employers believed the care to be outside the HCPs’ scope of practice) [27], (3) practices that restricted referrals [22], and (4) availability of alternative care providers [35]. Clinical practice considerations that influenced non-participation in ethically complex, legally available care included time, workload, and logistical concerns [27, 34, 35]. HCPs also considered their participation in care relative to their self-assessed competency and lack of knowledge [22, 27, 32, 34, 35], and considered whether another care provider could or should provide the care [21, 22, 25, 27, 29, 32]. This non-participation was explicitly noted in some articles as due to a lack of interest in the care area [25, 27], a lack of a desire to participate in care [29], or a belief that a specialist could provide better care [27]. Also influencing non-participation in ethically complex, legally available care was HCPs’ preference for other care options [32, 34], and their assessment that the precipitating condition could be managed in alternative ways [23]. Lastly, HCPs’ considered the circumstances that precipitated the patient’s care request [22–26, 33], and the availability of adequate care follow-up relative to their care participation [36].

## Discussion

### Main findings

While conscientious objection frequently dominates the discourse regarding HCPs’ non-participation in ethically complex, legally available care, the findings of this scoping review make clear that multiple factors beyond ethical, religious, or core moral beliefs [4] also influence HCPs’ non-participation. Non-participation in legally care available in a morally pluralistic, evolving care context that has significant physical, mental, emotional, and social implications was influenced by the emotional labour of care, the consideration of patient factors, HCPs' care preferences, practice logistics, and complexities, as well as the larger system within which HCPs work. Our findings align with a systematic review of nurses’ and midwives’ reasons for declining to participate in pregnancy termination [38], which identified moral, practical, religious, or legal reasons for objecting to care. Collectively, this highlights a need to distinguish between *conscientious objection to care* (when an HCP does not participate in care because doing so would be against “one or more of his deepest commitments” [39]) and *non-participation in care* due to reasons other than conscience (i.e., non-participation due to self-interest or professional integrity) [4]. This delineation is critical as the practice implications are different within each construct.

Non-participation in legally available care in a morally pluralistic, evolving care context with significant physical, mental, emotional and social implications that culminates in a conscientious objection is complicated. Existing codes of ethics frequently do not adequately capture the complex realities of practice, and the processes to disengage from care are ambiguous [40]. Nurses who had a conscientious objection reported feeling alone, uncertain, and stigmatized and that their objection felt futile due to a lack of meaningful professional support [41]. Thus, healthcare systems must mitigate the confusion and variability in conscientious objection policies [10] and address the disconnect between having a policy in situ, and the pragmatic, practical realities of enacting an objection [40]. The importance of this is paramount, considering the continuous advancements in healthcare and the resultant shifts in HCPs’ roles and responsibilities [42].

Non-participation in ethically complex, legally available care for reasons other than conscience requires authentic and continuous discussions among healthcare regulators, leadership, administrators, unit managers, and HCPs. These discussions will illuminate HCPs’ practice realities and support an enriched and nuanced understanding of the myriad of factors that are influencing non-participation. Self-assessed inadequate competence [22, 27, 32, 34, 35], time, workload and logistical concerns [27, 34, 35], uncertain policies [32], workplace practice limits [22, 27], and patient-related practice considerations [22–24, 26, 31, 33] all influenced HCPs’ non-participation in ethically complex, legally available care. Thus, policy clarification, removal of practice barriers and workplace practice limits, providing time and logistical support for care provision, and continuing education opportunities may positively support HCPs' participation. Additionally, professional regulators and associations must elucidate HCPs’ roles and obligations where duty, abandonment, and non-participation for reasons other conscience intersect. Elucidating these roles and responsibilities is crucial for all HCPs. However, this clarification is more acutely required for HCPs who practice in rural, remote, single-provider practices or in areas with limited referral options.

### Strengths and limitations

The inclusion of two of the largest practicing groups of healthcare providers (physicians and RNs) and the inclusion of multiple care areas were project strengths. An additional strength was the inclusion of articles where the care was legally available, thus removing the hypothetical factors influencing potential non-participation in care. Eight countries were represented in the included articles, and it was not possible to account for the diversity and impact of culture. Additionally, there may be different non-participation factors in different care areas or among the professional groups that were excluded from the project. Inclusion of specific ethically complex, legally available care areas may have excluded other care areas where care non-participation occurs. Further, utilization of identified databases may result in the exclusion of articles indexed in other databases, and the use of English only articles could have resulted in the exclusion of relevant articles in other languages.

### Areas of future research

With our article search and identification strategy, we discovered a significant body of literature (n = 10,664). However, when the results were limited to research articles of physicians and RNs within defined ethically complex, legally available care areas, the final number of articles markedly decreased. This suggests that although conscientious objection is frequently debated, explored, and deconstructed in the literature, there is significantly less research into the precipitating factors or underpinnings of HCPs’ care non-participation in ethically complex, legally available care. Equally important, as the discourse opens between conscientious objection and non-participation for reasons other than conscience, additional research into non-participation for reasons other than conscience is warranted. Of the included articles, the majority (14 out of 16) were concerning EOL and reproductive health (pregnancy termination and birth control) care areas. This suggests that research into the care areas of genetic testing, reproductive health and technology, and organ procurement may be underexplored. Lastly, future research could explore differences in the factors influencing HCP participation where care is legally available to those where it is illegal to distill the anticipated or hypothetical influencers of non-participation.

## Conclusion

As healthcare evolves and patient care options change, a robust understanding of the factors that influence HCPs’ who do not participate in ethically complex, legally available care is imperative. This understanding of the factors will further delineate conscientious objection and non-participation for reasons other than conscience as separate constructs such that HCPs are supported in a manner that is specific to the underlying factor influencing their participation.

## Data Availability

All data generated or analyzed during this study are included in this published article.

## Abbreviations

HCPs: Healthcare practitioners
RNs: Registered nurses
EOL: End-of-LIFE
MAID: Medical assistance in dying
CO: Conscience objection

## Declarations

### Protocol

Brown, J., Goodridge, D., Thorpe, L., & Chipanshi, M. (2018). Factors contributing to practitioner choice when declining participation in legally available care: A scoping protocol. BMJ Open, 8(8), e023901. 10.1136/bmjopen-2018-023901.
